# Examination of the Eating Behavior of the Hungarian Population Based on the TFEQ-R21 Model

**DOI:** 10.3390/nu12113514

**Published:** 2020-11-15

**Authors:** Zoltán Szakály, Bence Kovács, Márk Szakály, Dorka T. Nagy-Pető, Tímea Gál, Mihály Soós

**Affiliations:** Institute of Marketing and Commerce, Faculty of Economics and Business, University of Debrecen, H-4032 Debrecen, Hajdú-Bihar County, Hungary; szakaly.zoltan@econ.unideb.hu (Z.S.); kovacs.bence@econ.unideb.hu (B.K.); szakaly.mark@econ.unideb.hu (M.S.); gal.timea@econ.unideb.hu (T.G.); soos.mihaly@econ.unideb.hu (M.S.)

**Keywords:** eating attitudes, food consumption, behavior, food choice, TFEQ-R21, questionnaire

## Abstract

Several theories have emerged to study types of eating behavior leading to obesity, but most of the applied models are mainly related to food choice decisions and food consumer behavior. The purpose of this paper was to examine the eating attitudes of Hungarian consumers by applying the Three-Factor Eating Questionnaire (TFEQ-R21). The national representative questionnaire involved 1000 individuals in Hungary in 2019. Several multivariate statistical techniques were applied for the data analysis: exploratory and confirmatory factor analyses, multivariate data reduction techniques, and cluster analysis. This study successfully managed to distinguish the following factors: emotional eating, uncontrolled eating, and cognitive restraint. By using the factors, five clusters were identified: Uncontrolled Emotional Eaters; Overweight, Uncontrolled Eaters; Controlled, Conscious Eaters; the Uninterested; and the Rejecters; all of these could be addressed by public health policy with individually tailored messages. The empirical results led to rejection of the original Three-Factor Eating Questionnaire (TFEQ-R21), while the TFEQ-R16 model could be validated on a representative sample of adults, for the first time in Hungary.

## 1. Introduction

In recent decades, several theories have emerged to study types of eating behavior leading to obesity [1,2,3,4]. The research models applied in this field have mainly been related to food choice decisions and food consumer behavior.

Two main quantitative models have been highlighted for food choice decisions. One is a 36-statement questionnaire developed by Steptoe et al. [5] called the Food Choice Questionnaire (FCQ). The authors identified nine factors that reflect the motivations for an individual’s food choices: health, mood, comfort, palatability, naturalness, price, body weight control, familiarity, and ethical considerations. Fotopoulos et al. [6] claimed that empirical data do not support the robustness of the nine factors in the model, i.e., the applicability of the model is not the strongest. Renner et al. [7] also noted that while important motivational elements such as social and psychological aspects have been left out of the FCQ, the Eating Motivation Survey (TEMS) model already contains 15 basic types of motivation (factors). These are pleasure, habits, need and hunger, health, comfort, indulgence, traditional eating, naturalness, social life, price, visual temptation, body weight control, emotion regulation, social norms, and social image. According to the original model, 78 statements can be grouped into 15 factors [7], but after reducing the variables, the researchers also developed a 45-item model. Models for food consumer (eating) behavior include the Three-Factor Eating Questionnaire (TFEQ), a commonly used tool in the literature to assess eating behavior that considers the factors of cognitive restraint, emotional eating, and uncontrolled eating. The questionnaire, originally consisting of 51 items (factors), was developed by Stunkard and Messick [8], with each statement measured on a dichotomous scale. The TFEQ [8,9,10] was developed based on the following three previously existing tests: the Latent Obesity Questionnaire [11], the Eating Attitudes Test [12], and the Restraint Scale [13].

In recent decades, the understanding of eating behavior has been dominated by restraint or mitigation factor of restraint (R) [14]. Currently, there are three widely used restrictive eating questionnaires, i.e., the TFEQ-R, the Dutch Eating Behavior Questionnaire (DEBQ) [15], and the Revised Restraint Scale (RRS) [16]—the third one developed from an original experiment measuring restrictive eating [17]. External and emotional eating, while combined in the TFEQ in the construction of disinhibition, are assessed separately in the DEBQ and have been shown to be independently related to body weight [18]. Over time, the development of the TFEQ resulted in two additional factors, i.e., disinhibition (D) and hunger (H). The disinhibition factor (D) can be associated not merely with higher Body Mass Index (BMI) values and obesity but also with less healthy dietary choices. Regarding the hunger factor (H), it was also concluded that high scores, as was previously the case, could result in higher body weight [19,20]; this finding, however, was refuted by Bryant et al. [14], who argued that the hunger subscale was only rarely associated with a change in body weight. Of the three subscales, the least researched is the TFEQ-H, which was originally intended to measure susceptibility to hunger [21].

These scales were originally designed to measure long-term attitudes toward eating, which is why some authors refer to them as measurements of traits or characteristics (e.g., behavioral characteristics); see [14,22,23,24].

Several authors have attempted to create alternative versions of the model. Westenhoefer et al. [25] and Bond et al. [26] suggested that additional subscales could be formed from the three general TFEQ factors. Cognitive eating restraint can be further divided into subscales of rigid and flexible restraint. The former measures the adaptive dietary control strategy that does not result in eating disorders; the latter, on the other hand, focuses on the maladaptive dietary strategy likely to be associated with episodes of disinhibition (Westenhoefer [27] cited in Czeglédi et al. [28]). The disinhibition factor can be further divided into three subscales: habitual, emotional, and occasional propensity for eating in a disinhibited manner [26]. Ricciardelli and Williams [29] reported a three-factor model of restraint (R). The factors they identified were named as follows: emotional/cognitive dieting, knowledge of caloric content, and behavioral dietary control [4]. Allison et al. [30] developed a two-factor model (cognitive restraint and behavioral restraint) that, however, could not be later confirmed by statistical analysis. Consequently, the most important task for the widespread application of the TFEQ was to support the factor structure of the measuring tool, which generated a number of psychometric studies [4]. There are also abbreviated versions of the questionnaire consisting of fewer factors (statements or items), but the most common ones are the 21-item (TFEQ-R21) and the 18-item (TFEQ-R18) versions [9,10], which are most popular within the disciplines of psychology and sociology [31]. This is presumably supported by the fact that the 51-item version, which is also popular, has been claimed to be inapplicable to the study of different peoples and groups by several authors [9,32], i.e., it does not resonate with the original factor structure. Nonetheless, the original questionnaire has been adapted for a number of countries. For example, there are Thai [33], and Spanish versions [34], as well as a version applied to obese and overweight Chinese people living in Singapore [35]. The different versions are most often not even given a separate name, but cases such as the German version [8,36] and the DEBQ (Dutch) version mentioned above can be identified.

Some authors have sought correlations between the TFEQ and other questionnaires related to nutrition. James et al. [37] compared the three (R–H–D) 51-item scales of the TFEQ with a more recently published 16-item WREQ (Weight-Related Eating Questionnaire) model in the course of a one-year weight loss trial. The latter measures the scales of routine eating, compensatory restraint, and external and internal emotional eating. The WREQ combines elements of the TFEQ and the DEBQ [15] with a number of new questions added in order to assess two types of restraint (routine and compensatory) and two types of disinhibition (external and emotional). Thus, the WREQ aims to combine the strengths of both questionnaires. However, data for the authentication of the WREQ are currently limited. Duarte et al. [38] developed an 11-factor questionnaire (Inflexible Eating Questionnaire—IEQ) that can be interpreted as a practical implementation of the ideas of Westenhoefer et al. [25] on rigid and flexible cognitive eating control presented in this section. Linardon et al. [39] repeatedly referred to the author and colleagues mentioned above. Mention must be made of Chong et al. [35], who surveyed the TFEQ-51 to 444 Chinese participants based on four separate studies and scored according to different alternative versions of the TFEQ. Confirmatory factor analysis (CFA) and a goodness-of-fit test were performed to determine the most appropriate factor structure. Their findings suggested that the Niemeier disinhibition factors and the TFEQ-R18 factor structures are the most appropriate for a given population based on fit indexes. Of the three factors of the TFEQ-R18, only two (emotional and uncontrolled eating) showed good internal consistency, while none of the Niemeier D (disinhibition) factors [40] showed good internal consistency. Known-group validity showed that emotional eating and internal disinhibition were significantly associated with higher BMI values. Finally, the factor structure of the TFEQ-R18 was found to be the most appropriate and practical to measure the eating behavior of the overweight and obese Chinese population in Singapore. In addition to the TFEQ and the nutrition questionnaires directly related to it, there are also a number of validated questionnaires on nutrition for clinical and other uses.

Since eating behavior is influenced by the interactions of cognitive, affective, and behavioral factors [26], exploring and understanding the psychological aspects of eating attitudes can help to develop appropriate intervention and prevention programs.

Our research can be considered unique in several respects. First of all, the questionnaire was applied and validated to the entire adult population of a representative sample, not merely obese people. Thus, this research fills a scientific gap in this field. In addition to factor analysis, cluster analysis was also performed in the course of the research in order to identify consumer segments; this was also carried out by Soós [41] alone in Hungary on a representative sample, but that research was not validated for the whole population. In view of the above points, the objectives of the current research were as follows:

1. Determining the factor structure in Hungary based on the items of the TFEQ-R21 questionnaire.
2. Examining the applicability of the measuring device to assess the eating behavior of the entire adult Hungarian population.
3. Defining consumer segments (clusters) based on eating attitudes.

## 2. Materials and Methods

### 2.1. Sampling Method

Data collection was carried out in November 2019 by means of personal interviews, with interviews conducted at the respondents’ homes. The primary research was based on a national questionnaire-based survey representative of gender (Chi-square (χ2) (1) = 0.760; probability value (*p*) = 0.383) and age group (χ2 (5) = 0.421; *p* = 0.520). In the sampling process, representativeness was also ensured for regions (χ2 (6) = 6,997; *p* = 0.321) and settlement types (χ2 (2) = 3.409; *p* = 0.182), so their structure perfectly matched the quota set in advance by the Hungarian National Statistical Office (quota sampling). In the assigned settlements, a random walking method was used to ensure total randomness in selection. The essence of the method is that each interviewer was given a randomly selected starting address in the given settlement. From the starting address, in ascending order by house number, the interviewers began the questioning at the third house on the same side of the street, and then, if they were done there, they continued at the next third house. During the compilation of the sampling plan, it was also made sure that the interviewers should have no problem whether they were conducting the questioning in a district with detached houses or in a district with blocks of flats. Among the residents of the visited household, the person suitable for the interview was selected by using the so-called birthday key method. Hence, from among the residents of the households visited, those participants whose birthday was the closest to the date of the survey were selected for the interview. With this method, randomness was ensured only in each strata. The sample consisted of 1000 persons, since in Hungary the number of people in the age group examined is approximately 8 million [42], and with a 95% confidence level and a 5% margin of error (on the basis of Gill and Johnson [43]), the required sample size is 385 respondents. Consequently, the sample size was appropriate for examining the research objectives. Table 1 shows the percentage distribution of the socio-demographic groups of the individuals involved in the survey and the population composition according to the previously mentioned four factors.

### 2.2. Structure of the Questionnaire

The questionnaire was made up of two parts: the 21 statements of the TFEQ and 9 questions concerning socio-demographics (gender, age, highest level of education completed, marital status, legal status, net income, assessment of health awareness, body height, and body weight). The questionnaire was in Hungarian. The original English questionnaire had been translated into Hungarian by Czeglédi-Urbán [4], and that questionnaire was used by the authors after permission was requested and granted. Participants were asked to rate the 16 statements of the TFEQ-R21 questionnaire on a four-point Likert scale according to the extent to which they found themselves experiencing what was being described (1—definitely true; 2—mostly true; 3—mostly false; or 4—definitely false). Respondents were also able to enter a value of 0, which stood for the category “do not answer/do not know.” Of the 16 statements, 7 items were related to uncontrolled eating, 6 items were related to emotional eating, and the remaining three items were related to different aspects of cognitive restraint. The remaining 5 questions covered the following issues: avoiding the stocking of tempting foods, consuming less consciously, feeling hungry, and the extent of self-restraining behavior. Four of these items were 4-point questions, and one item was an 8-point Likert-type scale question [4]. The 5 questions were as follows:

1. How often do you avoid “stocking up” on tempting foods? (1—Almost never; 2—Seldom; 3—Usually; 4—Almost always; 0—Do not know)
2. How likely are you to make an effort to eat less than you want? (1—Unlikely; 2—A little likely; 3—Somewhat likely; 4—Very likely; 0—Do not know)
3. Do you go on eating binges even though you are not hungry? (1—Never; 2—Rarely; 3—Sometimes; 4—At least once a week; 0—Do not know)
4. How often do you feel hungry? (1—Only at mealtimes; 2—Sometimes between meals; 3—Often between meals; 4—Almost always; 0—Do not know)
5. On a scale from 1 to 8 (where 1 means no restraint in eating and 8 means total restraint), how would you rate yourself?

### 2.3. Data Analysis

In order to examine the research objectives, multivariate statistical tools were primarily used. First, exploratory factor analysis was performed on the model. The aim of the exploratory factor analysis (EFA) was to explore whether the pre-hypothesized factor structure appeared in our sample and whether we were able to measure the desired attitudes (factors that can be defined as latent variables). Then, we examined the reliability of the scales within the measurement model of the revealed latent variables using the Cronbach’s alpha index and the composite reliability index. The reliability test was followed by a CFA. The purpose of the CFA analysis was to prove the convergent validity, i.e., whether our empirical model fit the assumed model. Discriminant validity was tested according to the Fornell–Larcker criterion. For further examination, data reduction by principal component analysis (PCA) was performed separately on the latent variables in order to obtain latent variables free of cross-loadings. The segmentation was performed by cluster analysis, which consisted of two main steps: first, the number of clusters/segments was determined by hierarchical cluster analysis, and then the cluster analysis was carried out using the K-means method, in which the cluster means were determined by the applied program. In order to examine the clusters, cross-tabulation analysis and simple hypothesis tests were used. For CFA analysis, v3.5.0. of R Statistics in the RStudio editor was used (The R Foundation, Vienna, Austria), and all additional tests were performed in v23.0. of IBM SPSS Statistics (Armonk, New York, USA).

## 3. Results

### 3.1. Exploring Eating Attitudes

In the course of our research, we first examined eating attitudes by using the first 16 statements of the Three-Factor Eating Questionnaire. The descriptive statistical characteristics are illustrated in Table 2.

The findings indicated that only to a small extent did respondents feel eating triggered by a momentary emotional state (nervousness, depression, loneliness, sadness, tension, anxiety, etc.) to be true in their case. These factors showed the lowest standard deviation and coefficient of variation, but even in this way, a highly variable data set could still be observed. The results were around values above 3.00, which was confirmed by the negative skewness value (i.e., they considered the statements less able to truthfully describe themselves). These were followed by statements related to feelings of hunger and binge eating, all of which were rated above 3.00, i.e., respondents mostly did not consider them true when describing themselves. Consumers found the statements that could be related to conscious restraint and the balance of body weight to be the truest in their case. The tools used in the measurement model do not meet the requirements of a normal distribution, but this was not required by the methodology of the study. Numerous studies have shown that normal distribution is relatively rare in social sciences [46,47,48]. The methods used in this study were either nonparametric tests or robust enough in terms of the number of items to handle an abnormal distribution [49,50,51].

After the evaluation of the first 16 questions, the results of the answers to the last five questions are now presented. The first of these questions was to find out how often respondents avoid stocking up on tempting foods in their environment. The results are shown in Table 3.

The results indicated that the majority (62%) reported to rarely or never avoid tempting food, i.e., they like keeping such products in their homes or where they are currently staying. This suggests that almost two-thirds of the population have a need for these types of food. In contrast, the group of conscious consumers was only 10.3%—they are the ones who reported avoiding appetizing foods.

The next question dealt with the issue whether the respondents would make any effort to eat less than they would like to. Data on this are demonstrated in Table 4.

Similarly to the responses given to the previous question, it was also the less conscious behavior that dominated, as 60.7% of respondents said that they do not want to make any effort to eat less than they would like to. The proportion of those who reported being able to limit the amount of food they consume was merely 10%, while 28.7% of respondents were able to make some efforts.

After that, it was asked how often respondents ate something even if they were not hungry. Detailed information on this question is provided in Table 5.

The results showed that slightly more than half of respondents only reported eating when they are hungry, i.e., they avoid consuming larger amounts of food in case of satiety. On the other hand, the proportion of consumers with the answer “Rarely” could be considered high, i.e., one third of the population said that they tend to indulge even with a full stomach. It was found that 14.3% of the population is prone to excessive food consumption, and they represent the group of the undisciplined.

The next question was to find out how often respondents feel hungry. The most important findings are listed in Table 6.

Almost 40% of respondents reported getting hungry only at mealtimes, but a further 48.1% sometimes reported suffering from hunger even between meals, i.e., they may be prone to snacking. Only few of the respondents claimed being often hungry between main meals (12.5%), while the number of those in the “Almost always” category was extremely low (0.8%).

The last question concerned the self-restrictive behavior of respondents (Table 7). Respondents’ eating habits were analyzed on an eight-point scale, where a score of 1 meant no restraint in eating and a score of 8 stood for total restraint.

The results supported previous findings. The largest group comprised those who reported not showing restraint in eating at all. The group size was almost 25%. The five categories following the group of consumers who did not engage in self-restrictive behavior showed balanced proportions, while the lowest were those in categories 7 and 8 (8.4% and 2.9%, respectively).

The exploration of eating attitudes was performed by exploratory factor analysis. During the EFA, a priori and statistical inconsistencies were found in the model with the original 21 variables, and this was also confirmed by the CFA. In the case of five measurement variables, we observed too high cross loadings in the maximum likelihood (ML) procedure, while in the PCA procedure, the professional interpretation of the obtained latent variables caused difficulties. In order to solve this problem, we removed the five dissonant items from the model and continued the validation process on the measurement model narrowed down to 16 statements. During this process, the 16 statements in the questionnaire concerning eating attitudes were taken into account, with the help of which we could identify factors that characterize Hungarian consumers. The results in Table 8 indicate that in terms of eating attitudes, Hungarian consumers can be divided into three main groups (factors). In the analysis, we obtained a model with a high explanatory power of 66.12%. The first and the strongest factor is emotional eating, which represented 44.95% of the variance. The high factor weights suggest that the eating attitudes of Hungarian consumers are shaped by the value dimension to a large extent, and it is sharply separated from the other dimensions. By examining the skewness of the factor (skewness index), it could be concluded that the distribution is significantly skewed to the left (skewness = −1.24), i.e., Hungarian consumers do not consider this way of thinking true for themselves. The second factor is uncontrolled eating, which is associated with excessive appetite and eating. The relatively high factor weights indicate that the factors of uncontrolled eating are significantly different from the other factors. The factor was found to be skewed to the left (skewness = −0.61), i.e., uncontrolled eating is less typical of Hungarian consumers. The last factor is cognitive restraint, which includes the characteristics of self-regulatory behavior. High factor weights in this case, too, suggest that the dimension greatly shapes the eating attitudes of Hungarian consumers. The skewness of the factor to the left was also found to be pronounced here (skewness = −0.43). Consequently, respondents do not feel it true of themselves either, but of the three factors, this dimension is the most accepted.

### 3.2. Examination of the Applicability of the Model

Prior to performing the segmentation, the suitability of the model for the studies had to be tested, and this was carried out in three steps: the reliability of the measuring tool (in this case the scales) had to be explored, the internal validity of the empirical model had to be explored to see whether the measuring instruments actually measure the latent variable for which they were intended, and, finally, we had to check the discriminant validity between the measurement and latent units of the model, according to which the measurement units explain the associated latent variable more strongly than any other examined latent variable.

Reliability was examined with Cronbach’s alpha, the composite reliability (CR) index, and the McDonald’s omega, based on which the scales we applied can be considered reliable, and reliability cannot be increased by removing further items [52,53]. The results of the reliability test are summarized in Table 9. All the three reliability indicators showed that the measurement variables (scales) belonging to the latent variables of the model were reliable. There were larger or smaller differences between the indicators at the level of each latent variable, and this was due to the different methodology of the indicators; however, consistent results could be observed from the indicators when we compared the reliability of the latent variables. The emotional eating factor was by far the most reliable according to all three indicators, and the reliability of the cognitive restraint factor was the lowest—although in this case, reliability could also be considered acceptable based on all three indicators.

The fit of the model was tested by CFA. The measurement and structural parts of the model (hierarchical relationship between measurement and latent variables) were developed according to the EFA supporting our preliminary assumptions. Table 10 demonstrates the results of the CFA analysis.

Based on the results, the internal validity of the model can be accepted [54], which means that our empirical model fit the theoretical model. In the CFA analysis, the only concession we made was to allow the covariance between the measurement variables for a given latent variable in the calculations in all the cases where the modification index showed outliers.

The convergent validity of the applied model was examined with the AVE (average variance extracted) indicator and with the factor loadings belonging to each measurement variable. The values of the AVE indicator can be seen in Table 11. According to the criterion defined based on the work of Hair et al. [51], the value of AVE must be above at least 0.5 to meet convergent validity. In one case (uncontrolled eating), the value of the AVE indicator was only slightly higher than expected [51]; however, according to Lam [55], convergent validity may exist even when the value of CR meets the expected level (0.6), so the convergence of the measurement variables towards the latent variable was acceptable. According to the criterion by Hair et al. [51], convergent validity is satisfied.

Discriminant validity was examined based on the work of Fornell and Larcker [56] in accordance with the requirements they set; namely, we determined the AVE of each latent variable, which was compared with the correlation coefficients between the latent variables (note: factor loadings from data reduction were taken into account in the calculation of both the CR and AVE indicators). Table 11 demonstrates the results of the study on difference validity. The second column of the table shows the AVE index; the third, fourth, and fifth columns show the correlation coefficients; and the square root of the AVE index is shown in bold. If the square root of the AVE is compared with the individual correlation coefficients, the difference validity can be determined, i.e., the Fornell–Larcker criterion is satisfied. Right before performing the segmentation, the data reduction of the revealed latent variables was carried out by means of analyzing the principal components separately in order to eliminate the biasing effect of cross-loads. This was of particular importance, since the obtained factors were used for further multivariate analyses in which a one-step data reduction would have created an information gap that would impede the accurate analysis of the clusters.

### 3.3. Segmentation Based on Eating Attitudes

During segmentation, the segmentation criteria were the previously defined attitudes (factors) based on the model of eating attitudes: emotional eating, uncontrolled eating, and cognitive restraint. Once our data proved suitable for segmentation, we determined the number of clusters with a hierarchical cluster analysis and examined whether we had outliers. Since no outliers were found and the number of segments was determined in five clusters, we ran the cluster analysis using the K-means method, during which the determination of cluster means was performed by the algorithm. Based on the analysis of variance, the formed clusters differed significantly from each other (*p* < 0.01), so the result of the segmentation can be regarded as valid. Below, in accordance with the objectives of the research, a detailed description is given of each cluster.

1. Cluster 1—Uncontrolled Emotional Eaters

The proportion of the group was 10.4% (101 people) of all respondents. In this segment, women were slightly overrepresented (11.3%), and the youngest (18–29 years of age—13.0%) and older (50–59 years of age—12.3%; 60–69 years of age—13.2%) age groups dominated; 30–39-year-olds and 40–49-year-olds were severely underrepresented (8.4% and 8.6%, respectively). The group had a higher proportion of respondents with primary schooling as their highest level of education (in Hungary, primary school finishes at 14 years of age) (11.0%) and of high school graduates (11.1%). However, those with a higher education degree (8.9%) were few compared to the size of the cluster. The segment was characterized by a slight dominance of those with an average financial situation (just enough to make a living—10.7%), while households with the best income were underrepresented (they make a very good living and can also save—5.4%). They typically reported living in the capital (19.8%), yet the proportion of those living in a county town was underrepresented (5.9%). Health awareness was not a typical feature in this cluster; respondents who are not at all, or mostly not, health-conscious (20.7% and 14.4%, respectively) were strongly overrepresented, while those claiming to be very health-conscious were very few (7.8%). According to the self-reported body mass index, overweight and obese women predominated (13.5% and 11.5%, respectively).

Among the groups, they were the ones who felt several statements to be true descriptions of themselves. Of these, they agreed primarily with statements relating to emotional and uncontrolled eating. With regard to certain attitude factors, they were closest to cluster 3: they avoid certain foods because they are fattening. The findings suggest that their uncontrolled behavior is influenced to some extent by the desire to prevent obesity and overweight. This is also likely to be supported by the fact that the group was dominated by 18–29 year olds, for whom the primary concern was found to be maintaining body weight rather than health preservation.

2. Cluster 2—Overweight, Uncontrolled Eaters

The proportion of the group within the total sample was 12.4%, i.e., 120 respondents. The segment was strongly overrepresented by men (16.6%) and slightly dominated by 18–29 (14.2%) and 40–49-year-olds (14.4%). Those with primary schooling as their highest level of education (12.8%) and skilled workers (16.4%) were overrepresented in the segment, while those with a degree in higher education were strongly underrepresented (5.6%). The members of the cluster were more typically characterized by a bad financial situation than not. The cluster was typically made up of respondents living in the capital and in villages (15.7% and 16.7%, respectively), while the proportion of those living in smaller towns was low (6.9%). The cluster is typically not health-conscious, as the proportions of respondents who are not at all health-conscious and mostly not health-conscious were 24.1% and 19.5%, respectively. Based on the body mass index, obese men strongly dominated within the cluster (23.3%).

Their eating attitudes can be characterized by uncontrolled eating: when they see or smell a delicious food, they find it very difficult to keep from eating, even if they have just finished a meal. They are strongly exposed to sensory stimuli, so they are influenced by the taste, the sight, the smell, and altogether by the sensory properties of the products. They find it hard to control their hunger, and they often feel hungry, which can typically lead to uncontrolled eating behavior. When they start eating, they just cannot seem to stop. The behavioral characteristics described above lead to overweight and obesity.

3. Cluster 3—Controlled, Conscious Eaters

With regard to its size, this was the second largest cluster of the five (22.8%, i.e., 220 respondents). By gender, women were strongly dominant (28.5%), while men were underrepresented (16.4%). Regarding age group, those aged 30–39 (26.6%) and those over 70 (24.7%) were overrepresented, while the proportions of those aged 40–49 (21.4%) and those aged 50–59 (20.5%) were slightly smaller compared to the size of the cluster. It was high school graduates and, mostly, respondents with a higher education degree that predominated in the segment (23.5% and 36.3%, respectively), while those with primary schooling (19.3%) and skilled workers (18.7%) were underrepresented. Within the group, households with a higher income dominated (make a living but can save little—25.1%; make a very good living and can also save—36.5%), while the proportion of those on average income was low (just enough to make a living—17.7%). According to the type of settlement, those living in the capital (24.8%) and those living in villages (24.0%) were overrepresented. The group is strongly health-conscious—the proportions of those considering themselves mostly and very health-conscious were significantly higher compared to the size of the group (43.2% and 48.0%, respectively). In contrast, those who are not at all health-conscious and mostly not health-conscious were strongly underrepresented (8.0% and 6.3%, respectively). Regarding body mass index, the proportion of low and proper weight women appeared remarkable in the group (27.6%), but those of women considering themselves overweight and obese were also high (29.7% and 27.9%, respectively).

The group can be characterized by controlled eating and self-regulatory behavior. They try to take small helpings in order to control their weight, and they avoid certain food because they are fattening. Regarding the latter, they are similar to cluster 1. Among all the clusters, they are the most conscious in limiting how much they eat during meals. Emotional and uncontrolled eating is typical of them only to a very small extent, and they can also resist sensory impulses. This is the cluster where we can find women who consider themselves overweight or actually are so (self-assessment); consequently, they do everything they can to restore their proper body weight. As seen above, women with a proper body weight were also more represented in this cluster, but they consciously maintain their own body weight.

4. Cluster 4—The Uninterested

This cluster comprised the third largest group within the sample (22.4%, i.e., 216 respondents). Within the cluster, men were slightly overrepresented (23.3%), with 30–39-year-olds (29.2%) and 60–69-year-olds (24.5%) dominating by age. In terms of education, the group could be considered balanced, though those with a higher education degree were slightly overrepresented (24.2%). The cluster was fully proportionate regarding subjective perception of income, with all income layers represented in accordance with the sample size. According to the type of settlement, those living in smaller towns were strongly overrepresented (31.5%), while the proportion of those living in the capital was quite low in the cluster (12.4%). The cluster was dominated by the less health-conscious groups (not health-conscious at all—25.3%; mostly not health-conscious—31.0%; both health-conscious and not—25.1%; mostly health-conscious—16.9%; and completely health-conscious—10.0%).

No outliers could be observed in any attitude statements. They seemed typically uninterested compared to the other clusters. The responses “mostly true” and “mostly false” were given for all the statements, i.e., awareness, uncontrolled eating and emotional eating are only moderately characteristic of them. A sense of neutrality, indifference, and disinterest lies behind this phenomenon, which could mainly be due to negligence. Their health behavior—as seen above—can rather be characterized by risk behavior.

5. Cluster 5—Rejecters

The cluster was the largest of the five segments (31.9%, i.e., 308 respondents). Within the group, men slightly predominated (34.1%), whereas the age groups were dominated by those aged 40–49 (32.1%), 50–59 (35.6%), and the elderly over 70 (44.2%). The presence of those with primary schooling (34.9%), skilled workers (32.2%), and high school graduates (33.2%) was more typical in the cluster, while those with a degree in higher education were underrepresented (25.0%). The segment had a higher proportion of those living in poorer financial conditions than of wealthier respondents (sometimes not enough to make a living—34.2%; just enough to make a living—34.5%; make a very good living and can also save—27.0%). The majority of the group reported living in county towns (40.5%). The health awareness based on self-assessment could be considered average in the group (both health-conscious and non-health conscious—40.3%). Men and women of low and proper weight (36.3% and 37.2%, respectively), as well as overweight men (38.4%), were overrepresented in the cluster.

In contrast to segment 4, the responses given by the members of this cluster were “mostly false” or “definitely false” for all the statements. This group could be characterized by a completely instinctive eating attitude; they do not attach a great deal of importance to meals or they can be described as showing disinterest or rejection. They are definitely not emotional eaters, as all the statements connected to this domain were sharply rejected by them. The rejection of uncontrolled eating is also strong, so they are not motivated by pleasure either when choosing food. They came closest to cluster 2 in terms of conscious restraint and self-regulatory behavior, i.e., they are not likely to keep up conscious nutrition in order to avoid obesity. This is probably due to the fact that this cluster included women and men with ideal body weights in the highest number; consequently, there is no strong motivation here to lose weight.

## 4. Discussion

The three-factor structure of the original TFEQ-R21 was confirmed by the confirmatory factor analysis regarding uncontrolled eating, emotional eating, and cognitive restraint. The reliability of the internal consistency of the questionnaire (Cronbach’s alpha coefficient) was 0.927 for the emotional eating factor, 0.829 for the uncontrolled eating factor, and 0.806 for the cognitive restraint factor. According to the results of the conducted study, the version of the 21-item factor structure (TFEQ-R21) adapted to the Hungarian population showed an adequate internal consistency, as well as convergent and discriminant validity.

Czeglédi and Urbán [4] conducted a survey among Hungarian university students to test the TFEQ-R21 model, ultimately surveying 262 university students in 2008. As a result of their research, they managed to validate the original TFEQ-R21 model (with 21 statements), but their results were certainly limited by the fact that the sample examined was not nationally representative and the number of items was significantly lower than in the present research. The three eating attitudes revealed in their research were consistent with the findings of the current study, although the rearrangement of certain measurement elements could be detected (e.g., “Sometimes when I start eating, I just can’t seem to stop.”).

In their study in 2016, Keller et al. [31] tested the TFEQ-R16 model among 919 individuals, and although their research was not clearly representative of gender, they found that the model narrowed down to 16 statements was suitable for examining the three-factor eating attitudes. Confirmatory factor analysis was not performed during their studies and convergent and difference validity were not examined, but their results suggested that the narrowed TFEQ-R16 may provide more suitable results. The results of Keller et al. [31] were closely related to the findings of the current study—the factors formed, the factor charges, and the reliability of results were very similar; only one difference could be detected: the rearrangement of a single item (“Sometimes when I start eating, I just can’t seem to stop.”) could be established.

Kavazidou et al. [57] tested the factor structure of the TFEQ-R18 model on a 495-person sample, concluding that the 16-statement model (TFEQ-R16) may be more suitable for the Greek population to study the three-factor eating attitudes model, although their research may have also been limited by the generalizability of the results. Furthermore, their research revealed that even the 18-statement model could provide a four-factor result, which may confirm the findings of the present study in that if the three-factor eating attitude in the original model (TFEQ-R21) is to be studied, the 16-statement model would reflect a clearer solution and yield a more transparent result.

In Hungary, Szabó et al. [58] were able to validate 12 items of the 21-item model for the entire population. They were as follows: six items of emotional eating, three items of uncontrolled eating, and three items of cognitive restraint. It was found that emotional eating had a greater role in the eating behavior of the entire adult population than cognitive restraint. In contrast, we were able to validate 16 items including seven items for emotional eating, six items for uncontrolled eating, and three items for cognitive restraint.

Regarding the 21-item questionnaire, it is generally acknowledged that regardless of the group in the study or the population surveyed, the three factors listed above have been confirmed in almost all the cases. The field of application of the 21-item TFEQ has been extremely wide, including the following research subjects: children on a diet and their mothers (e.g., [59]), adolescents (e.g., [60]), the overweight and people of normal build (e.g., [61]), university students (e.g., [4,62]), minors and children (e.g., [34]), and Generation Y (e.g., [31,63]). Occasionally, but rarely, the general population has also been tested (e.g., on a non-representative sample of 486 people [38]). Moreover, the 21-item version has already been used in the medical field (e.g., gastroenterology; see [64]), as have the 18-item and the 51-item versions.

The current study determined consumer profiles in a representative way based on the eating attitudes of the Hungarian adult population by using cluster analysis. This is probably the first study to cover the entire population, not merely a particular socio-consumer segment such as the obese. It can also be considered a gap filler in the sense that although the study of eating styles is still very popular today, very few studies have focused on segmenting consumers based on their eating styles.

However, the cluster analysis generated from the model has also been addressed by several other Hungarian authors (e.g., [58,63]), although not all of them exclusively used the TFEQ.

Most of the research studies using cluster analysis have been able to distinguish three consumer segments (e.g., [31,59,62,63]), while others, (e.g., [58]) similarly to us, could identify five clusters based on the TFEQ. Having surveyed a sample of 1000 people, Szabó et al. [58] outlined the following five factors: (1) uncontrolled emotional eaters, (2) tense dissatisfied eaters, (3) uninterested eaters, (4) overweight impulsive eaters, and (5) conscious eaters. Four of these segments (1, 3, 4, and 5) were identical in content to the segments we have described. However, the segment of rejecters identified in our research has different characteristics than the tense dissatisfied group described by the authors of [58].

The clear advantage of the current research is that the authors of the present study were the first in Hungary to validate the Three-Factor Eating Questionnaire on a sample that is large and representative of the Hungarian adult population. Overall, having compared the results of previous research, it can be concluded that numerous versions of the TFEQ model with different items are being used in practice, but several cases have confirmed the relevance of the set of questions containing 16 statements.

## 5. Conclusions

The results of this study indicate that Hungarian consumers feel that eating influenced by their current emotional state is typical of them only to a very limited extent, and by their own admission, they are not characterized by immoderation and loss of control either. The statements that could be related to conscious restraint and weight balance were considered by them to be the truest of themselves. This consumer perception, however, seems to contradict the results of the cluster analysis. Eating attitudes could be classified into three distinct factors, i.e., emotional eating, uncontrolled eating, and cognitive restraint. The three factors exist in parallel in the mindset of the adult population. Based on this, it can be concluded that the eating behavior of the Hungarian population can be well measured with the TFEQ. Only a quarter-to-one-fifth of the adult Hungarian population can be characterized by controlled, conscious eating, while the remaining 75%–80% can be classified as the uninterested, the rejecters, or the emotional and uncontrolled eaters. In the future, it will be an important task for public decision-makers and the corporate sector to substantially improve consumer awareness of nutrition. It will be necessary to develop intervention strategies and programs that differentiate the forms, the tools, and the content of communication based on the characteristics of the presented clusters. The central idea is the necessity to communicate with groups of controlled, conscious eaters in a different way than with the uninterested, dismissive, and uncontrolled emotional eaters. The results obtained during the research will hopefully enrich the international literature while simultaneously providing a basis for comparisons for further research. The findings of the study suggest that the TFEQ-R16 measuring device can be used not only among the obese but also among the general population—in our case, the entire adult Hungarian population—to analyze eating behavior.

In conclusion, the results of the current research are novel in the sense that the TFEQ-R16 model was validated on a representative sample of the appropriate size for the first time in Hungary, although, based on our empirical results, the application of the original model (TFEQ-R21) had to be rejected. We explored the presence and quality of the three eating attitudes examined among the Hungarian population, and the segmentation of the population based on the three eating attitudes can also be considered a new result.

## Figures and Tables

**Table 1:** Distribution of the sample according to the most important background variables (*N* = 1000) and population composition according to representative variables.

Label	Sample Distribution		Population Distribution ^1^
	Count	%	%
Male	471	41.1	47.8
Female	529	52.9	52.2
18–29 years	169	16.9	17.2
30–39 years	161	16.1	16.0
40–49 years	196	19.6	19.6
50–59 years	152	15.2	15.1
60–69 years	163	16.3	16.3
70 years	159	15.9	15.8
Budapest	181	18.1	17.9
Other town	550	55.0	52.6
Village	269	26.9	29.5
Western Transdanubia	100	10.0	10.1
Central Transdanubia	109	10.9	10.8
Southern Transdanubia	94	9.4	9.0
Northern Great Plain	148	14.8	14.8
Central Hungary	298	29.8	31.0
Northern Hungary	119	11.9	11.5
Southern Great Plain	132	13.2	12.7
Primary school	109	10.9	
Vocational school	394	39.4	
High school	364	36.4	
Higher education	133	13.3	
Can live on it very well and can also save	78	7.8	
Can live on it but can save little	392	39.2	
Just enough to live on but cannot save	427	42.7	
Sometimes cannot make ends meet	74	7.4	
Have regular financial problems	9	0.9	
Not known/No answer	20	2.0	

Note: ^1^ Source of data: [44,45].

**Table 2:** Statistical indicators of eating attitudes (*n* = 1000).

Attitude Statements	Statistical Indicator			
	Mean ^1^	Standard Deviation	Coefficient of Variation %	Skewness
When I feel nervous, I try to calm myself by eating.	3.31	0.96	28.94	−1.222
When I get depressed, I feel like eating.	3.30	0.94	28.39	−1.190
When I feel lonely, I console myself by eating.	3.30	0.99	29.88	−1.235
When I feel blue, I often overeat.	3.29	0.97	29.54	−1.196
When I feel tense or upset, I often feel like I need to eat.	3.28	0.95	28.84	−1.140
When I feel anxious, I find myself eating	3.23	1.01	31.49	−1.032
I get so hungry that my stomach often seems like a bottomless pit.	3.21	1.00	31.25	−1.026
I am always hungry, so it is hard for me to stop eating before I finish the food on my plate.	3.20	0.98	30.5	−1.020
Sometimes when I start eating, I just can’t seem to stop.	3.13	0.98	31.25	−0.807
I am always hungry enough to eat at any time.	3.09	0.97	31.42	−0.810
When I see a real delicacy, I often get so hungry that I have to eat right away.	3.07	0.92	29.90	−0.633
When I see or smell a delicious food, I find it very difficult to keep from eating, even if I have just finished a meal.	2.92	0.95	32.64	−0.479
I do not eat some foods because they make me fat.	2.91	1.13	38.69	−0.572
I consciously hold back at meals in order not to gain weight.	2.89	1.07	37.06	−0.490
Being with someone who is eating often makes me hungry enough to eat too.	2.86	0.99	34.72	−0.409
I deliberately take small helpings as a means of controlling my weight.	2.82	1.09	38.68	−0.430

Note: ^1^ Results are assessed on a scale from 1 to 4, where 1 stands for “Definitely true” and 4 corresponds to “Definitely false.”

**Table 3:** The distribution of responses to the question: “How often do you avoid “stocking up” on tempting foods?” (*n* = 1000).

Response Options	Distribution of Responses	
	Count	%
Almost never	307	30.7
Seldom	313	31.3
Usually	270	27.0
Almost always	103	10.3
Do not know	7	0.7

**Table 4:** The distribution of responses to the question: ‘How likely are you to make an effort to eat less than you want?’ (*n* = 1000).

Response Options	Distribution of Responses	
	Count	%
Unlikely	386	38.6
A little likely	221	22.1
Somewhat likely	287	28.7
Very likely	99	9.9
Do not know	7	0.7

**Table 5:** The distribution of responses to the question: “Do you go on eating binges even though you are not hungry?” (*n* = 1000).

Frequency	Distribution of Responses	
	Count	%
Never	526	52.6
Rarely	328	32.8
Sometimes	116	11.6
At least once a week	27	2.7
Do not know	3	0.3

**Table 6:** The distribution of responses to the question: “How often do you feel hungry?” (*n* = 1000).

Frequency	Distribution of Responses	
	Count	%
Only at mealtimes	379	37.9
Sometimes between meals	481	48.1
Often between meals	125	12.5
Almost always	8	0.8
Do not know	8	0.8

**Table 7:** The distribution of responses to the question: “How would you rate yourself on a scale from 1 to 8, where 1 means no restraint in eating and 8 means total restraint?” (*n* = 1000).

Category	Distribution of Responses	
	Count	%
1	236	23.6
2	115	11.5
3	106	10.6
4	181	18.1
5	138	13.8
6	112	11.2
7	84	8.4
8	29	2.9

Note: 1—No restraint in eating at all, 8—Total restraint.

**Table 8:** The results of the exploratory factor analysis of the Three-Factor Eating Questionnaire.

Statements	Factors		
	Emotional Eating	Uncontrolled Eating	Cognitive Restraint
When I feel nervous, I try to calm myself by eating.	0.824		
When I feel blue, I often overeat.	0.777		
When I get depressed, I feel like eating.	0.776		
When I feel tense or upset, I often feel like I need to eat.	0.766		
When I feel anxious, I find myself eating	0.744		
When I feel lonely, I console myself by eating.	0.721		
Sometimes when I start eating, I just can’t seem to stop.	0.497		
When I see or smell a delicious food, I find it very difficult to keep from eating, even if I have just finished a meal.		0.731	
When I see a real delicacy, I often get so hungry that I have to eat right away.		0.678	
I am always hungry so it is hard for me to stop eating before I finish the food on my plate		0.582	
I am always hungry enough to eat at any time.		0.563	
Being with someone who is eating often makes me hungry enough to eat too.		0.521	
I get so hungry that my stomach often seems like a bottomless pit.		0.501	
I do not eat some foods because they make me fat.			0.777
I deliberately take small helpings as a means of controlling my weight.			0.757
I consciously hold back at meals in order not to gain weight.			0.706

Extraction method: maximum likelihood; rotation method: varimax rotation; rotation converged in 6 iterations; Kaiser-Meyer-Olkin (KMO) = 0.923 (excellent); Bartlett: (approximate chi square) 9357.054; Significance probability (Sig.) 0.000; communalities: 0.318–0.699; cumulative explained variance: 66.118; *n* = 1000.

**Table 9:** The reliability of the measuring tools applied.

Factors	Cronbach’s Alpha	Composite Reliability	McDonald’s Omega
Emotional eating	0.927	0.980	0.929
Uncontrolled eating	0.829	0.895	0.819
Cognitive restraint	0.806	0.701	0.809

Source: authors’ own calculations.

**Table 10:** The reliability of the measuring tools applied.

Indicator	Acceptance Region	Empirical Results
χ^2^		289
df.		71
sig.	<0.05	<0.001
CFI	>0.9	0.970
GFI	>0.9	0.960
AGFI	>0.9	0.935
RMSEA	<0.07	0.056
SRMR	>0.9	0.962

Note: χ^2^ = Chi-square; df. = degrees of freedom; sig. = significance probability; CFI = comparative fit index; GFI = goodness-of-fit index; AGFI = adjusted goodness-of-fit index; RMSEA = root mean-square error of approximation; SRMR = root-mean-square residual. Source: authors’ own calculations and [54].

**Table 11:** Results of difference validity.

Latent Constructs	AVE	Latent Constructs		
		Emotional Eating	Uncontrolled Eating	Cognitive Restraint
Emotional eating	0.702	0.837		
Uncontrolled eating	0.526	0.657	0.725	
Cognitive restraint	0.732	0.311	0.108	0.856

Note: AVE = average variance extracted. Source: authors’ own calculations.
